# Narrowing numerous nascent RNA-sequencing strategies

**DOI:** 10.1093/plcell/koad243

**Published:** 2023-09-20

**Authors:** Nora Flynn

**Affiliations:** Assistant Features Editor, The Plant Cell, American Society of Plant Biologists; Department of Botany and Plant Sciences, University of California, Riverside, CA 92507, USA

How can we study RNA that is in the process of being synthesized? For over half a century, researchers have developed diverse approaches to isolate and sequence nascent RNA and reveal layers of transcriptional control that are invisible when capturing only mature RNA (Wissink et al. 2019). For example, nascent RNA sequencing (RNA-seq) can provide insight into polymerase movement, transcription rates, and unstable or noncoding RNAs.

Historically, there are three major classes of nascent RNA-seq protocols. First, there are approaches that rely on nuclear run-on assays, such as global run-on-sequencing (GRO-seq) (Core et al. 2008). During the run-on assay, a nucleotide analog is incorporated into actively transcribed RNA to allow immunoprecipitation of nascent RNA. Second, nascent RNA can be captured by immunoprecipitating RNA polymerase II (Pol II), such as in plant native elongating transcript sequencing (pNET-seq) (Zhu et al. 2018). Finally, after chromatin isolation, Pol II can remain bound and chromatin-bound (CB) RNA can be sequenced (Weber et al. 2014). Each of these methods has strengths and weaknesses, making it difficult to choose the best approach for specific research needs.

In this issue of *The Plant Cell*, **Min Liu and colleagues** (Liu et al. 2023) systematically compare the major nascent RNA-seq approaches—GRO-seq, pNET-seq, and CB RNA-seq—and provide detailed guidance for selecting a method. The authors also modify existing approaches to introduce two new strategies that reduce undesirable RNAs: chromatin native elongation transcript sequencing (ChrNET), where chromatin is used as the starting material for pNET-seq, and 3′CB RNA-seq, where library preparation captures the 3′ end of nascent RNA and RNase H digestion removes abundant noncoding RNAs. Each approach is evaluated for reproducibility, cost, and ability to detect transcriptional activity, RNA Pol II stalling, and splicing (see Fig.).

**Figure.**
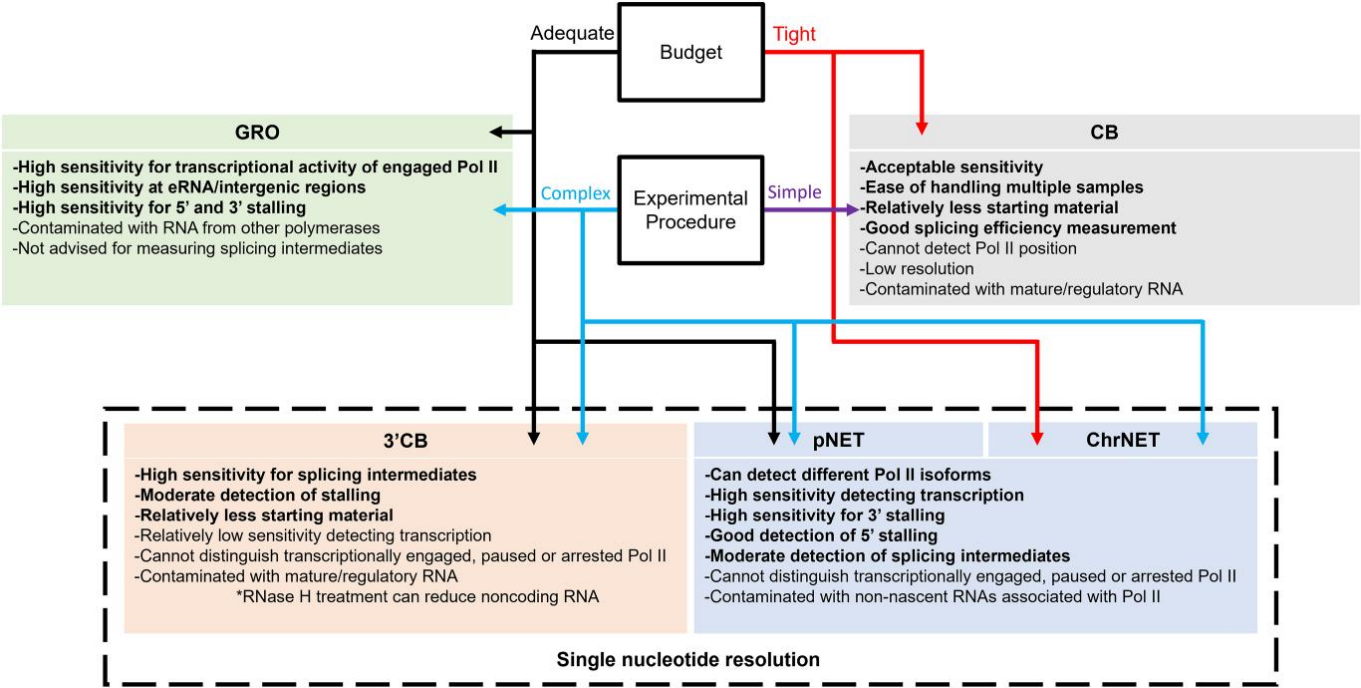
Guide for selecting a nascent/CB RNA-seq strategy. Adapted from Liu et al. (2023), Figure 8 and Table 1.

The authors first tested reproducibility and sensitivity. Each method was highly reproducible within replicates. Across methods, GRO, pNET, and ChrNET were particularly well correlated, indicating that these methods may be more accurate at measuring transcriptional activity. pNET and ChrNET were also more sensitive and could identify the most actively transcribed genes, with the genes identified by ChrNET being the most reproducible. Similarly, for detecting long noncoding RNA, pNET and ChrNET were the most sensitive, but other methods rivaled these approaches in identifying active miRNA genes. Overall, ChrNET consistently matched or rose above competing methods for detecting nascent RNA and is cost-effective with reduced reads from rRNA and plastid RNA.

For RNA Pol II stalling, all methods can detect RNA Pol II position except for CB RNA-seq, where RNA is fragmented before library preparation. The 5′ stalling detection was largely repeatable among methods, but 3′ stalling was less correlated, although pNET, ChrNET, and GRO still show positive correlations. However, it should be noted that pNET, ChrNET, and 3′CB might detect some terminating or arrested RNA Pol II, while GRO might detect more paused polymerases that have actively transcribed during the run-on assay.

Finally, pNET and 3′CB could detect co-transcriptional splicing intermediates, with 3′CB showing particularly high sensitivity and accuracy. 3′CB revealed nearly double the number of predicted 5′ splice sites compared to pNET. Over 85% of these sites came from annotated sites. CB RNA-seq was also useful to identify candidates for recursive splicing, where two consecutive splicing steps are required to completely remove an intron. Overall, 3′CB and CB RNA-seq were the most effective tools to investigate splicing intermediates and splicing efficiency, respectively.

When studying nascent RNA, there are numerous approaches to choose from, and each of these approaches shines in unique areas. Not all methods were tested here, although the authors provide some additional comparisons of other popular approaches by using published datasets. In summary, the in-depth trials and explanations provided offer useful advice when selecting an approach for nascent RNA sequencing.
